# Solid-Phase Microextraction

**DOI:** 10.3390/molecules25020379

**Published:** 2020-01-17

**Authors:** Constantinos K. Zacharis, Paraskevas D. Tzanavaras

**Affiliations:** 1 Laboratory of Pharmaceutical Analysis, Department of Pharmaceutical Technology, School of Pharmacy, Aristotle University of Thessaloniki, GR-54124 Thessaloniki, Greece; 2 Laboratory of Analytical Chemistry, School of Chemistry, Faculty of Sciences, Aristotle University of Thessaloniki, GR-54124 Thessaloniki, Greece

Undoubtedly, sample preparation is one of the most important steps in the analytical process. It is estimated that approximately 60% of workload, time, and cost are devoted to sample preparation. Among other modern sample preparation techniques, solid-phase microextraction (SPME) is a state-of-the-art, solvent-free technology that was developed by the research group of Pawliszyn [1] in 1989. Due to the fact of its versatility, reliability, low cost, and sampling convenience (i.e., on-site sampling), SPME has been widely used in combination with separation techniques (e.g., LC, GC, and CE) in academic research and routine analyses. Up to now, SPME has been successfully utilized in numerous applications in various scientific fields. As a result of its impact, a search in Scopus revealed almost 2000 publications (e.g., research articles, reviews, book chapters) reporting research/applications using SPME.

The present Special Issue includes sixteen outstanding contributions covering the latest research trends and applications of SPME. The advantages of SPME were exploited by the research group of Koziel [2] in their research work “Development of Time-Weighted Average Sampling of Odorous Volatile Organic Compounds in Air with Solid-Phase Microextraction Fiber Housed Inside a GC Glass Liner: Proof of Concept”. One of the primary goals of the work was to fabricate a rugged SPME-based sampler that can be deployed for longer periods in remote locations. Acetic acid was utilized as a model compound, since it is the most abundant VOC in any animal facility including swine farms. The researchers concluded that Car/PDMS material provided superior performance compared to other materials.

An analogous topic is published by Kenessov et al. [3] in their research regarding “Optimization of Time-Weighted Average Air Sampling by Solid-Phase Microextraction Fibers Using Finite Element Analysis Software”. An SPME model was developed with both absorptive and adsorptive fibers located inside a protective needle using a finite element analysis-based software. This model was utilized to determine the potential sources of quantification inaccuracies of the time-weighted average sampling of VOCs in ambient air. Various SPME parameters were investigated and optimized. Using the modeling results, alternative sampling geometries were proposed.

The research group of Queiroz [4] present “A Dual Ligand Sol-Gel Organic-Silica Hybrid Monolithic Capillary for In-Tube SPME-MS/MS to Determine Amino Acids in Plasma Samples”. A hybrid organic-silica monolithic capillary column with amino and cyano groups was fabricated and evaluated as an extraction device for in-tube SPME. The manufactured material was characterized using various techniques including scanning electron microscopy, Fourier transform infrared spectroscopy, nitrogen sorption experiments, among others. The in-tube SPME was utilized for the extraction of amino acids and neurotransmitters from plasma samples obtained from schizophrenic patients. The detection of the analytes was carried out using tandem mass spectrometry.

An automated SPME method is proposed by Dugheri et al. [5] in their article entitled “High-Throughput Analysis of Selected Urinary Hydroxy Polycyclic Aromatic Hydrocarbons by an Innovative Automated Solid-Phase Microextraction”. A commercially available xyz robotic apparatus was employed to facilitate direct-immersion SPME in combination with GC-QqQ-MS for the determination of hydroxy-based metabolites of polycyclic aromatic hydrocarbons in urine samples. These analytes were used as biomarkers of internal doses to access recent exposure to polycyclic aromatic hydrocarbons. An on-fiber derivatization protocol using *N*-tetr-butyldimethylsilyl-*N*-methyltrifluoroacetamide was followed in order to enhance the gas chromatographic properties (i.e., volatility) of the analytes.

Herraez-Hernandez and co-workers [6] in their article “Analysis of Contact Traces of Cannabis by In-Tube Solid-Phase Microextraction Coupled to Nanoliquid Chromatography” reported a powerful tool based on in-tube SPME in combination with nano-LC for the quantitation of contact traces of drugs (e.g., cannabis). A set of cannabinoids was tested on various surfaces involving aluminum foil, office paper, hand skin, etc. The main difficulty in the analysis of contact traces of drugs is the low amount of available sample that is often only visible through microscopy. A relatively simple extraction protocol was employed after sampling using cotton swabs.

Research data regarding the permeation of chemical compounds through skin is provided by the group of Baynes [7] in the publication “Skin Permeation of Solutes from Metalworking Fluids to Build Prediction Models and Test a Partition Theory”. A membrane-coated SPME fiber was utilized simulating skin permeation. The work aimed at the investigation of the permeation of 37 analytes through the membrane under certain conditions associated with skin exposure to several fluids (mineral oil, polyethylene glycol 200, synthetic oil, etc.) widely used in the metalworking industry.

Headspace SPME is an interesting alternative offering several advantages. These features were exploited by Chen et al. [8] in their research work entitled “Volatile Terpenes and Terpenoids from Workers and Queens of *Monomorium chinense* (Hymenoptera: Formicidae)”. The objective of this study was to identify certain terpenes and terpenoids, determine their glandular origins, and study the effect of diet on terpene composition. The obtained data helped the authors to find out whether de novo terpene and terpenoid synthesis occurs in this species of ant. 

Bialowiec et al. [9], in their study “Quantification of VOC Emissions from Carbonized Refuse-Derived Fuel Using Solid-Phase Microextraction and Gas Chromatography-Mass Spectrometry”, present quantitative data from the analysis of VOCs from carbonized refuse-derived fuel using headspace SPME combined with GC-MS. The analyzed samples were generated from the torrefaction of municipal waste. A commercially available three-component SPME fiber was used for the analytes’ extraction. The authors concluded that the VOC emitted from the torrefied samples were different from that emitted by other types of pyrolyzed samples, produced from either different types of feedstock or under different pyrolysis conditions.

The biological changes of the Mediterranean fruit fly during mating procedures were the main target of the work of Ren et al. [10] entitled “Application of Direct Immersion Solid-Phase Microextraction (DI-SPME) for Understanding Biological Changes of Mediterranean Fruit Fly (*Ceratitis capitata*) During Mating Procedures”. This report investigates the feasibility of using DI-SPME high-resolution metabolism for the profiling of fruit fly tissues at various stages of adulthood. The obtained results were statistically treated using principal component analysis.

Characterization of the aromatic profile of mango germ sperm using headspace SPME is reported by the research group of Wang [11] in the paper “Analysis of the Volatile Profile of Core Chinese Mango Germplasm by Headspace Solid-Phase Microextraction Coupled with Gas Chromatography-Mass Spectrometry”. A standard SPME protocol was followed for the extraction of aroma volatiles from the samples. The authors found that there were quantitative and qualitative differences in the volatile compounds among Chinese mango cultivars.

A sensomics approach combined with principal component analysis was exploited by Ye and co-workers [12] in their work entitled “Discrimination of Aroma Characteristics for Cubeb Berries by Sensomics Approach with Chemometrics”. The aroma profiles of cubeb berries were evaluated by different extraction approaches involving hydro-distillation, simultaneous distillation/extraction, and SPME followed by GC-MS-olfactometry. The experimental parameters affecting the performance of SPME were studied and optimized. Almost 90 volatile compounds were identified in the studied samples. 

Koziel and colleagues [13], in their communication “Evaluation of Volatile Metabolites Emitted In Vivo from Cold-Hardy Grapes during Ripening Using SPME and GC-MS: A Proof-of-Concept”, reported the exploitation of SPME coupled with GC-MS in order to evaluate the volatile metabolites produced from cold-hardy grapes. Special glassware in conjunction with SPME was employed for the non-destructive sampling of biogenic volatiles emitted by the grape cluster. 

A two-dimensional GC × GC-TOF-MS method was developed by Chen et al. [14] for the analysis of volatile compounds in pears. Their results are published in the article entitled “Analysis of Volatile Compounds in Pears by HS-SPME-GC×GC-TOFMS”. After the optimization of the SPME conditions, the authors finally identified 241 compounds in the tested samples consisting of esters, alcohols, and aldehydes. Cluster analysis was used for the treatment of the results. 

An application of headspace SPME is reported by Lyczko et al. [15] in the article “HS-SPME Analysis of True Lavender *(Lavandula angustifolia* Mill.) Leaves Treated by Various Drying Methods”. The main objectives of this work was to determine the volatile profile composition of true lavender leaves and also the effect of three drying protocols applied. The analyses were carried out using GC-MS. An interesting finding was that the drying process may decrease the share of camphor while increasing the share of linalool and linalyl acetate which are the most desirable components in true lavender aroma.

An interesting application of headspace SPME is contributed by Cecchi and colleagues [16] in “GC-MS and HS-SPME-GC×GC-TOFMS Determination of the Volatile Composition of Essential Oils and Hydrosols (By-Products) from Four *Eucalyptus* Species Cultivated in Tuscany”. In this report, a preliminary characterization of the volatile profile of samples obtained from various *Eucalyptus* species was carried out. After SPME sampling, GC×GC-TOF/MS was employed for fingerprint analysis. 

Last but not least, Videau et al. [17], in the article “Profiling Volatile Constituents of Homemade Preserved Foods Prepared in Early 1950s South Dakota (USA) Using Solid-Phase Microextraction (SPME) with Gas Chromatography-Mass Spectrometry (GC-MS) Determination”, presented the development of a novel analytical method based on SPME sampling in argon-filled gas sampling bags with direct GC-MS determination. The main scope of this work focused on the volatile profiling of 31 homemade preserves prepared in South Dakota (USA) during the period 1950–1953.

Generally, publishing a Special Issue always puts considerable pressure on the authors and reviewers. The Guest Editors of this Special Issue would like to thank them for their timely responses and constructive comments and hope that this issue will prove to be full of substance for the readers.
